# Epigenetics of Myotonic Dystrophies: A Minireview

**DOI:** 10.3390/ijms222212594

**Published:** 2021-11-22

**Authors:** Virginia Veronica Visconti, Federica Centofanti, Simona Fittipaldi, Elisa Macrì, Giuseppe Novelli, Annalisa Botta

**Affiliations:** 1 Department of Biomedicine and Prevention, Medical Genetics Section, University of Rome “Tor Vergata”, Via Montpellier 1, 00133 Rome, Italy; virginia.veronica.visconti@uniroma2.it (V.V.V.); federica.centofanti@gmail.com (F.C.); simona.fittipaldi@uniroma2.it (S.F.); elisa.macri314@gmail.com (E.M.); novelli@med.uniroma2.it (G.N.); 2 IRCCS (Institute for Treatment and Research) Neuromed, 86077 Pozzilli, Italy; 3 Department of Pharmacology, School of Medicine, University of Nevada, Reno, NV 89557, USA

**Keywords:** myotonic dystrophies (DMs), epigenetics, methylation, chromatin remodeling, microRNAs (miRNAs)

## Abstract

Myotonic dystrophy type 1 and 2 (DM1 and DM2) are two multisystemic autosomal dominant disorders with clinical and genetic similarities. The prevailing paradigm for DMs is that they are mediated by an *in trans* toxic RNA mechanism, triggered by untranslated CTG and CCTG repeat expansions in the *DMPK* and *CNBP* genes for DM1 and DM2, respectively. Nevertheless, increasing evidences suggest that epigenetics can also play a role in the pathogenesis of both diseases. In this review, we discuss the available information on epigenetic mechanisms that could contribute to the DMs outcome and progression. Changes in DNA cytosine methylation, chromatin remodeling and expression of regulatory noncoding RNAs are described, with the intent of depicting an epigenetic signature of DMs. Epigenetic biomarkers have a strong potential for clinical application since they could be used as targets for therapeutic interventions avoiding changes in DNA sequences. Moreover, understanding their clinical significance may serve as a diagnostic indicator in genetic counselling in order to improve genotype–phenotype correlations in DM patients.

## 1. Introduction

Epigenetics is the study of heritable and stable changes in gene expression that do not directly alter the DNA sequence [1]. Epigenetic modifications are reversible, but they rarely remain through generations in humans, despite persisting through multiple cycles of cell replication [2]. The contribution of epigenetic changes in the pathogenesis of monogenic diseases is well established, with a myriad of papers focused on DNA repeat expansions diseases [3,4,5]. Expanded unstable repetitions establish the complex and dynamic networks linked with epigenetic components, which could explain their instability and tendency to expand [5,6]. This review provides an updated overview of the literature data describing the epigenetic effects of the dynamic mutations underlying myotonic dystrophies (DMs), which are the most common forms of muscular dystrophies in adults. DMs are progressive multisystem repeat expansion disorders with clinical and genetic features in common. To date, the epigenetic post-transcriptional changes contributing to DMs pathogenesis are largely described, with either confirmed or controversial results. There are at least three main epigenetic mechanisms: changes in DNA methylation (DNAme), chromatin remodeling and expression of regulatory microRNAs (miRNAs). Research over the last decade has shown that the variability in clinical phenotype and age of onset of DM1 patients may be related to an *in cis* effect on the DNAme of CpG islands located within the *DMPK* gene. Chromatin dynamics are also increasingly recognized to influence both the instability of repeat expansions and the expression of genes adjacent to the DM1 locus. Indeed, it has been shown that long repeated stretches induce the formation of heterochromatin, which can spread to neighboring DNA regions [7]. A global miRNAs deregulation in DM tissues has also been reported first in muscle and then in blood leading to the identification of noninvasive diagnostic and prognostic disease biomarkers. Based on these observations, it is clear that the field of epigenetics is already generating novel potential prognostic and therapeutic avenues for DMs and other largely incurable diseases.

## 2. Genetics of Myotonic Dystrophies

**Figure 1:**
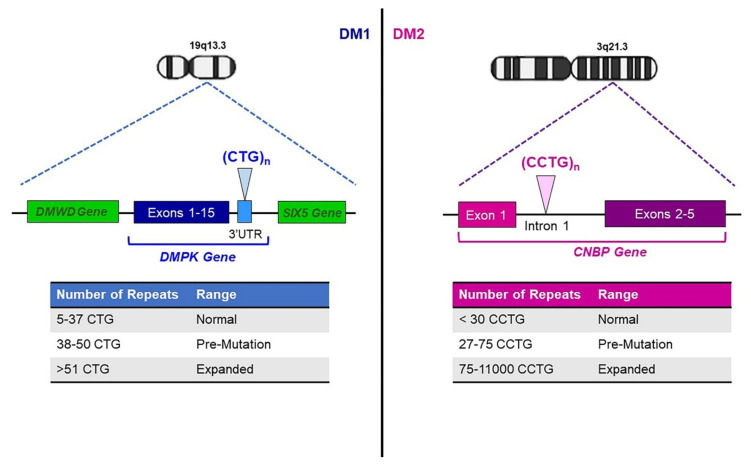
Genetics of myotonic dystrophies. DM1 is caused by a (CTG)_n_ expansion in the 3′ UTR of the *DMPK* gene on chromosome 19q13.3. Healthy individuals carry (CTG)_5-37_, *DMPK*-expanded alleles contain (CTG)_≥51_. DM2 is caused by a (CCTG)_n_ expansion in the first intron of *CNBP* gene on chromosome 3q21.3. Healthy alleles contain (CCTG)_<30_, whereas expanded alleles contain 75-11,000 copies of CCTG repetitions.

DMs are autosomal dominant muscular disorders, characterized by myopathy, myotonia and multisystemic involvement [8]. Despite clinical similarities, DMs include two genetically distinct diseases requiring different diagnostic and management strategies. Myotonic dystrophy type 1 (DM1, Steinert’s disease; MIM #160900) is caused by a (CTG)_n_ expansion in the 3′ UTR of the *DMPK* gene (MIM *605377) on chromosome 19q13.3, while myotonic dystrophy type 2 (DM2; MIM #602668) is caused by a (CCTG)_n_ expansion in the first intron of *CNBP* (previously *ZNF9*) gene *(*MIM *116955), on chromosome 3q21.3 (Figure 1) [9,10]. The estimated prevalence of DMs is about 1/8000, although DM1 seems to occur more frequently than DM2, with exceptions in Germany, Finland and Czech Republic, where both diseases are almost equally represented [11,12,13]. The clinical common features are caused by a gain-of-function RNA mechanism in which expanded CUG and CCUG repeats generate nuclear foci resulting in sequestration of RNA-binding proteins and dysregulated splicing of premessenger RNAs [14,15,16]. In DM1 patients, (CTG)_n_ expansion ranges from 51 repeats to several thousand with interruptions of the CTG array reported in about 3–5% of DM1 patients [17,18,19]. The repeat lengths of 38–50 CTG_s_ are considered premutation alleles, which show increased instability toward larger pathologically expanded repetitions [20,21]. Contrary to the (CTG)_n_ repeat, the (CCTG)_n_ tract in *CNBP* gene is part of a complex repeat, with polymorphic regions in the configuration (TG)_n_(TCTG)_n_(CCTG)_n_(NCTG)_n_(CCTG)_n_ [22]. Healthy alleles contain fewer than 30 (CCTG)_n_ repetitions, whereas expanded alleles contain 75–11,000 copies of uninterrupted (CCTG)_n_ [21] (Figure 1). Unstable premutated alleles range between 27 and 75 (CCTG)_n_ and have still uncertain clinical significance [23,24]. The DM2 phenotype typically arises in adulthood, and no congenital form of the disease has been reported [25]. A moderate correlation seems to exist in DM1 between CTG length, age of onset and disease severity. Generally, late-onset DM1 individuals show mild symptoms with less than 150 (CTG)_n_ repeats, and adult-onset DM1 patients carry between 100 and 1000 (CTG)_n_, while congenital and childhood-onset DM1 individuals have more than 1000 repeats and a more severe phenotype [25,26]. However, the genotype–phenotype correlation in DM1 is still far from being completely understood. In this context, the discovery of expanded *DMPK* alleles containing variant non-CTG repeats (VRs) within the repeated array is a contributory factor for explaining the phenotypic variability of the disease [17,18,27,28]. In fact, DM1 patients carrying “variant” (CTG)_n_ expansions seems to be less severely affected, with a delayed disease age at onset and a slower progression of the DM1 symptoms [17,18,19,29,30]. In addition, somatic expansions during the lifetime of DM1 individuals represent a substantial bias because they confound attempts to measure the exact length of the expanded *DMPK* allele, leading to over- or under-estimation. An accurate measurement of the progenitor allele length (ePAL) represents, to date, the best predictor of age at symptoms onset, providing a correlation between repeat size and phenotype [26,31,32]. The meiotic instability of the (CTG)_n_ repetitions leads to genetic anticipation where increased size of DM1 mutation and a more severe phenotype have been reported in affected individuals across generations [33]. Conversely, the DM2 mutation does not have a strong bias towards intergenerational expansion, and correlations between disease severity and the repeat size are relatively feeble [9,25]. The DM2 mutation usually contracts in the next generation, and this could explain some distinct features of the disease such as the absence of a congenital form, the lack of genetic anticipation and the later onset of symptoms [8].

## 3. Methylation of *DMPK* and *CNBP* Genes

**Figure 2:**
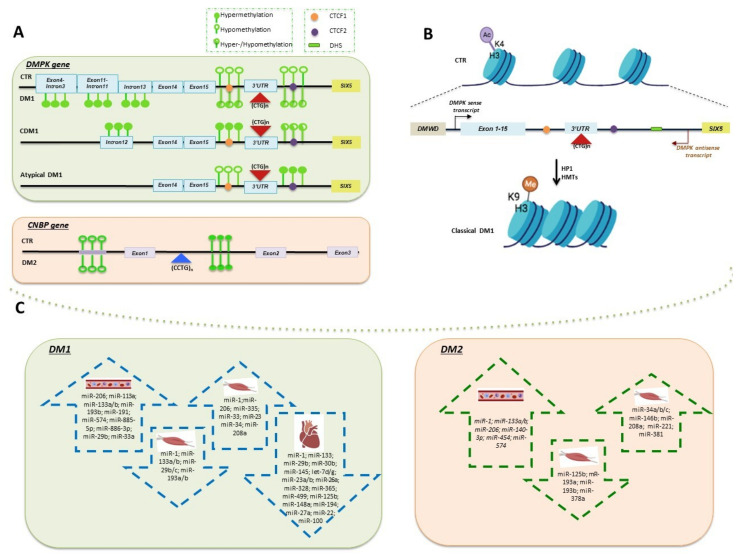
Epigenetic mechanisms in DMs pathogenesis. (**A**) Schematic representation of DNAme status in classical DM1, CDM1, atypical DM1, DM2 and CTR individuals. The two putative CTCF binding sites upstream (CTCF1) and downstream (CTCF2) of the CTG repeat at the *DMPK* locus are pictured as orange and violet circles, respectively. Empty green circles represent unmethylated CpG sites; filled green circles represent hypermethylated sites and the half-full green circles indicate both hyper- and hypo-methylated status. (**B**) Schematic drawing of chromatin remodeling of DM1 locus in CTR and classical DM1. Histone PTMs changes upon (CTG)_n_ expansion reflect a less active chromatin environment. A schematic drawing of DM1 locus is also represented, indicating the expanded CTG array with flanking CTCF sites, the DNAse hypersensitive site (DHS) and the *DMPK* sense and antisense transcripts. (**C**) Most relevant deregulated miRNAs in skeletal muscle, heart and blood from DMs patients. Upregulated and downregulated miRNAs are included in blue and green dashed arrow-shaped boxes for DM1 and DM2 samples, respectively.

DNAme is a dynamic and reversible change in gene activity or function that plays a key role in regulating gene expression by different mechanisms. The most relevant studies on DNAme in both DM1 and DM2 are summarized in Table 1. The contribution of this epigenetic mechanism in the DM1 pathogenesis is related to the different clinical subtypes of the disease: congenital (CDM1), childhood-onset, classic adult-onset and late-onset [26]. CDM1 is the most severe form, associated with large expansions and almost exclusive maternal transmission [34]. A methylation-sensitive restriction enzymes analysis of the DM1 locus provided the first evidence of DNA hypermethylation in intron 12 upstream of the (CTG)_n_ repeat in CDM1 patients [35] (Figure 2A). The identification of specific CTCF binding modulator sites (CTCF1 and CTCF2) flanking the CTG array and forming an insulator element between *DMPK* and *SIX5* genes has been the starting point for further functional analyses. CTCF is a zinc finger protein, and its binding to an insulator mediates the inhibition of promoter-enhancer interactions [36]. CpG methylation of CTCF1 prevents the binding of the insulator protein CTCF, affecting chromatin dynamics [37,38]. By contrast, DNAme at the CTCF2 site is highly variable in different tissues of the same DM1 patient, and CTCF binding to this region appears controversial [34,39,40]. Aberrantly methylation in CDM1 may be responsible for the loss of insulator activity between the *SIX5* enhancer and the *DMPK* promoter, resulting in higher *DMPK* expression levels and in a more severe clinical feature [37]. A methylation profiling revealed more than 10% methylation of two or more CpG sites at both CTCF sites in 20 CDM1 patients with maternal inheritance, confirming that the methylation pattern could represent a signature of CDM1 (Figure 2A) [34]. However, later studies performed in different tissues showed that abnormal methylation is not restricted to CDM1 individuals, but it is rather variably associated with the presence of the DM1 expansions [35,40]. Heart, liver and cortex showed high-to-moderate *DMPK* methylation levels, whereas cerebellum, kidney and skeletal muscle were devoid of methylation in DM1 adults [40]. Methylation levels also decreased between DM1 fetuses and adults, and only upstream sequences of the (CTG)_n_ repetitions appeared methylated, whereas the downstream sequences resulted methylation-free. Interestingly, DM1 mice showed the same upstream profile as DM1 human tissues, with a slight methylation pattern of the downstream sequence as well [40]. Additional analyses showed that the tissue-specific epigenetic features of *DMPK* neighboring genes could regulate the expression of *DMPK* itself. The myogenic hypermethylation at the intron 3/exon 4 border in *DMPK* is associated with strong decreases in CTCF binding but was shown to increase CTCF binding within the 3′ end of *DMWD* (a gene located near the 5′ of DMPK) and 0.5 kb upstream of the *DMPK* (Figure 2A). This evidence suggests that *DMWD* could act as an enhancer that stimulates *DMPK* gene upregulation in a tissue-specific manner [41]. Studies on DM1 in vitro cellular models have also contributed to elucidating the role of epigenetics in DM1 disease pathogenesis. The analysis of DM1-affected human embryonic stem cells (hESCs) allowed to identify a differentially methylated region (DMR) located 900 bp upstream the CTGs in intron 13 of *DMPK* (Figure 2A), with a hypermethylated pattern associated with lower *SIX5* expression. Interestingly, abnormal methylation was already established in the undifferentiated state and was exclusively acquired by larger expansions (>300 CTG repeat copies) [39]. The methylation analysis was extended to eight loci surrounding the (CTG)_n_ tract, five upstream and three downstream, in blood from 90 DM1 patients. Importantly, for the first time, a correlation between increased DNAme levels in exon and intron 11 of the *DMPK* gene (Figure 2A) and the modal length of CTG repeats was described. This evidence strengthens the hypothesis that the epigenetic changes and the modal length of CTG repeats are related to each other [42]. More recently, DNAme was also studied in DM1 patients carrying VRs alleles with non-CTG tract, including CCG, CTC or GGC motifs. A methylation-sensitive high-resolution melting (MS-HRM) analysis was performed in a study cohort including both DM1 patients with “pure” CTG expansion and DM1 “atypical” patients carrying VRs. In accordance with previous results, this analysis revealed the hypermethylation of DNA regions 5′ to the (CTG)_n_ repetitions in DM1 patients with congenital or childhood-onset form, significantly associated with maternal transmission. Interestingly, expanded *DMPK* alleles containing non-CTG repeats showed an opposite hypermethylation pattern restricted to the 3′ end of the (CTG)_n_ array [27] (Figure 2A). The increased DNAme levels at 3′ UTR downstream region in “atypical” DM1 patients compared to DM1 patients with “pure” CTG tract were further confirmed in later studies, as described below in the text [28,42]. VRs were also characterized in *DMPK* premutated alleles segregating in a three-generation Italian family [43]. The length and structure of the *DMPK* premutation remained stable over time, through either paternal or maternal transmissions. CpG analysis of DNA sequences flanking the (CTG)_n_ array did not show an aberrant pattern of DNAme associated with the presence of VRs within *DMPK* premutated alleles [43]. What about the stability of DNAme as epigenetic signature over time in the same individual? The answer to this question comes from a longitudinal study analyzing non-CDM1 patients, including VRs patients, sampled both at the time of diagnosis and up 27 years [44]. Higher methylation in the blood of DM1 patients than in healthy controls was reported and associated with the maternal inheritance of the disease. Nevertheless, the upstream CpG sites were not useful as biomarkers of DM1 since only the downstream sites showed a significant methylation difference in the patients compared to the control group. In line with previous results, VRs were associated with increased downstream methylation. Interestingly, the DNAme levels upstream and downstream of the (CTG)_n_ repeat remained stable over time in blood, indicating the potential use of this epigenetic signature as a biomarker for DM1 [44]. All the studies described so far are strictly focused on DM1, and the in cis effect of (CCTG)n expansion on the DM2 locus is still largely unknown. To date, only one study analyzed the methylation status of *CNBP* gene in whole blood and skeletal muscle tissues from DM2 patients [45]. The hypomethylation of CpG sites upstream and hypermethylation of CpG sites downstream of the (CCTG)_n_ expansion was observed either in DM2 patients and healthy individuals (Figure 2A), with no significant differences. Moreover, *CNBP* gene expression seems to be independent from the methylation pattern of the DNA regions analyzed. These preliminary evidences suggest the existence of molecular mechanisms other than epigenetics involved in the pathogenesis of DM2 [45].

## 4. Chromatin Remodeling of DM1 Locus

The chromatin organization and accessibility across the genome reflect a network of interactions through which promoters, enhancers, silencers, insulators, chromatin-binding factors and transcription factors cooperatively modulate gene expression. In the past few years, identifying epigenetic processes involved in human diseases, in particular processes influencing chromatin remodeling and thereby gene expression, is acquiring a fundamental importance. Indeed, their dysregulation leads to aberrant chromatin remodeling, which has been seen to be associated with several diseases [46]. An increasing number of studies are showing that in triplet expansion diseases, unstable trinucleotides not only contribute to disease progression but are also involved in aberrant chromatin remodeling [7]. For instance, in the case of Fragile X syndrome (MIM #300624) [47] and a Friedreich’s ataxia (MIM #229300) [48], the expanded trinucleotide repeat (CGG and GAA, respectively) is associated with hypermethylation and heterochromatinization, causing the silencing of *FMR1* (MIM *309550) and *FXN* (MIM *606829) genes, respectively. Few data are currently available on the chromatin environment of the DM1 locus in relation to expanded CTG-repeat traits. The human DM1 locus is a ~45 kb region at chromosome 19q13.3, which encompasses not only the *DMPK* gene but also two neighborhood genes: *SIX homebox 5* gene (*SIX5;* MIM *600963) and *dystrophia myotonica WD repeat–containing* gene (*DMWD;* MIM *609857) [49]. The main mechanism of chromatin remodeling at DM1 locus identifies the (CTG)_n_ expansion as a strong nucleosome-binding site that could potentially alter chromatin structure leading to regional effects on the expression of multiple genes. This is due to the fact that the expansion overlaps not only the 3′end of *DMPK* but also the 5′promoter region of the neighboring *SIX5 gene* [50]. More in-depth studies have reported a change in local chromatin structure in muscle and skin fibroblasts from DM1 patients, in which chromatin shows a decreased sensitivity to DNAse in a region approximately 500 bp downstream to the (CTG)_n_ expansion, indicating a conversion of the region to heterochromatin (Figure 2B) [51]. The loss of the hypersensitive site specifically in the *DMPK*-expanded allele results in less accessibility of proteins to DNA, thus explaining the mechanism through which the expression of *DMPK* itself and neighboring genes could be impaired. Indeed, Frisch et al. demonstrated that the loss of sensitivity to *PvuII* digestion in DM1 alleles results in a reduction of either *DMPK* and *SIX5* mRNA, whereas no changes in the expression of *DMWD* are observed [52]. Histone post-translational modifications (PTMs) also play an important role in the epigenetic regulation of chromatin structure, therefore influencing the transcriptional processes. The acetylation and methylation of the N-terminal histone tails are the most common PTMs able to define chromatin structure and domains through the recruitment of protein and complexes with specific enzymatic activities [53]. As mentioned before, (CTG)_n_ repeats are part of a CTCF-dependent insulator located between *DMPK* and *SIX5* genes [37], and this could place the CTCF binding sites in an internucleosomal accessible position at DM1 locus [37], supporting the idea of a more compact chromatin structure in this region [54]. Later studies showed that CTCF limits the extent of the *DMPK* antisense transcript, which emanates from the *SIX5* regulatory region adjacent to the downstream (CTG)_n_ repeat [55]. Furthermore, CTCF constrains the H3-K9 methylation to the nucleosome associated with the (CTG)_n_ repeat, whereas the expanded allele in CDM1 is associated with the loss of CTCF binding, spread of heterochromatin and regional CpG methylation (Figure 2B) [38]. Chromatin immunoprecipitation (ChIP) studies confirm a less active chromatin environment around the CTG expanded tract characterized by a decrease in H3K9/14Ac enrichment (a marker of active chromatin) at both CTCF binding sites. Moreover, the active histone modification H3K4me3 is replaced by an enrichment in H3K9me3 (a marker of transcriptionally repressed regions) upon the expansion of the (CTG)_n_ repeat (Figure 2B) [7]. Taken together, these data reveal a clear distinction in chromatin structure between the wild type and the expanded *DMPK* alleles. By contrast, Sorek *et al.* revealed a strong signature of active chromatin of the DM1 locus, such as an enrichment in H3K4me3, H3K27ac and H3K4me1. This reflects a common feature for polyQ disease-related genes that share a very active profile enriched in active marks and depleted in repressive marks in unaffected individuals. It has been suggested that an open chromatin state could be the cause of genomic instability, and in this sense, the heterochromatinization process can be considered as a protective mechanism from acquiring more mutations [56]. Finally, recent studies revealed a link between 3D genome folding and several expansion disorders in which genes are placed at the boundaries between topologically associating domains (TADs) that are highly enriched on CpG islands, suggesting that repeat expansions could disrupt TADs structure, resulting in a reorganization of the genome topology [57]. In order to test whether the DM1 locus shows an altered 3D chromatin conformation, Ruiz Buendía and collaborators performed 4C- and ChIP-Seq analyses of DM1 patient-derived lymphoblastoid cell lines (LCLs). What emerged from this study is that chromatin interactions and CTCF occupancy do not change upon (CTG)_n_ expansions, in fact CTR and DM1 LCLs show a similar 4C-and ChIP-Seq profile [58]. These results are in contrast to the previously reported effects of expanded CTG repeats on chromatin conformation. This discrepancy can be explained by the different cell type analyzed in this study, as differences in genetic background of LCLs could have a confounding effect on the chromatin interactions made at expanded DM1 locus [58]. It is therefore clear that further research is needed to assess how chromatin remodeling can contribute to DM1 pathogenesis. Currently, no studies are available on the influence that expanded (CCTG)_n_ repetitions may exert on the chromatin structure of the DM2 locus.

## 5. miRNAs-Based Mechanisms of Epigenetic Regulation in DMs

miRNAs are 18–24 nucleotide ncRNAs molecules that play a crucial role as modulators of epigenetic architecture inducing changes in gene expression levels post-transcriptionally by mRNA cleavage or translation repression [59]. Each miRNA can regulate multiple target mRNA transcripts, and each mRNA transcript can be regulated by different miRNAs, thus creating a complex regulatory network in numerous biological processes [60]. miRNAs display a different distribution across human tissues, and most of them are expressed in a highly tissue-specific manner [61]. Furthermore, miRNAs have been found in extracellular space or circulation (c-miRs), suggesting their putative function as cell-to-cell communications signals during various physiological and pathological conditions [62]. Interestingly, miRNAs can affect enzymatic effectors involved in epigenetic modulation such as enzymes, which take part in methylation-mediated silencing and chromatin remodeling; in turn, they can be also targeted for the epigenetic machinery [63]. Different studies have demonstrated a global miRNAs deregulation both in DM1 and in DM2 tissues. The most relevant studies focused on the role of miRNAs in DM diseases are summarized in Table 2 and are described in this section in relation to the biological sample analyzed: muscle or hearth (tissue-specific) and blood (circulating miRNAs).

### 5.1. Tissue-Specific miRNAs Deregulated in DMs

It is widely recognized that a set of miRNAs, also called “myomiRs”, are exclusively (muscle-specific miRNAs) or preferentially (muscle-enriched miRNAs) expressed in striated muscle [77]. They control signaling pathways known to regulate muscle health and development [77]. Distinctive patterns of myomiRs expression in different types of muscular dystrophies have been described [78]. Their release by myofibers can be induced by acute muscle damage [79], suggesting their key regulatory role in the pathological pathways leading to muscle dysfunctions. The distinctive features of muscular symptoms in DMs [80] and the critical role played by myomiRs in the striated muscle [77,81] have addressed the study of myomiR as prognostic and diagnostic biomarkers of DMs. Different papers have investigated myomiRs expression levels in muscle and cardiac tissues from DM1 patients, often leading to contradictory results. The first miRNAs profiling was performed on *vastus lateralis* biopsies of 7 DM1 patients with comparable expansion size and 4 control subjects. The expression of myomiRs, *miR-1*, *miR-133 a/b*, *miR-206* and *miR-108a/b/c* and of two predicted CTG-repeat binding miRNAs (*miR-103* and *miR-107*) has been profiled, showing an increase in *miR-206* expression level (Figure 2C) [64]. Afterwards, Perbellini et al., [65] performed a miRNAs analysis on *biceps brachii* from 15 DM1 patients indicating the cellular mislocalization of *miR-206*, *miR-1* and *miR-133b* (Figure 2C) [65]. The authors also found an increase in *miR-1*, *miR-335* expression level and a downregulation of *miR-33* and *miR-29 b/c*, with no changes in *miR-206* expression level. Interestingly, the study of *miR-1* and *miR-29* predicted target genes revealed a potential link to DM1 physiopathology with genes involved in myogenic differentiation, muscle cell excitability and splicing, which have been found significantly to be upregulated in DM1 patients [65]. As expected, *miR-29* downregulation was associated with an upregulation of *TRIM63*, *DIABLO*, *RET* and *TGFB3*—all genes known to be induced in atrophic myofibers [65]. The unexpected upregulation of *miR-1* predicted targets involved in muscle development, arrhythmia and splicing, has been explained by hypothesizing that the mislocalization of *miR-1* could lead to a loss of function resulting in a de-repression of its targets [65]. Further investigations evaluated *miR-1* expression level in different types of skeletal muscle biopsies [66,67,68] and heart tissues [69,70] from DMs patients, confirming the significant *miR-1* downregulation in DMs muscle tissues (Figure 2C) [50,51,52,53,54,55]. Although to date only two studies have investigated miRNAs pattern changes in heart tissues from DMs patients, their results are important to find out the relationship between miRNAs deregulation and cardiac defects in DMs. Both RNA processing [69] and transcription defects [70] have been proposed as putative mechanisms of miRNAs deregulation. In one study on heart tissues from DM1 (n = 5) and DM2 (n = 2) patients compared to 8 healthy subjects, miR-1 downregulation was linked to the MBNL1 dysfunction as cytoplasmic regulator of *pre-miR-1* processing. Indeed, the authors indicated a mechanism by which the depletion of free MBNL1 by expanded (CUG)_n_ or (CCUG)_n_ repeats allows *LIN28*-mediated uridylation of *pre-miR-1*, which resulted in a decreased expression of mature *miR-1* (Figure 2C) [69]. Moreover, the functional studies on *miR-1* predicted targets revealed a direct correlation between *miR-1* loss and an increased expression of gap junction (Gja1) and calcium channel (Cacna1c) proteins, whose deregulation is consistent with the cardiac dysfunctions observed in DMs patients [69]. Nevertheless, Kalsotra et al., [70] did not find any correlation between the loss of MBNL1 or gain of CELF1 activity and altered miRNAs expression in DM1. Therefore, they suggested that the decreased level of *miR-1* and other 22 *mi*RNAs is attributable to a reduction of the transcriptional program controlled by myocyte enhancer factor-2 (Mef2). Again, we must point out the paucity of studies addressing miRNAs deregulation in DM2 samples [69,76]. One of these studies, as discussed above, was performed in cardiac tissue from a mixed population of patients with DM1 and DM2 and demonstrated that the *miR-1* processing deregulation was associated with heart defects in both type 1 and type 2 DMs [69]. The study of miRNAs in DM2 muscle tissues identified 11 deregulated miRNAs, including seven upregulated (*miR-34a-5p*, *miR-34b-3p*, *miR-34c-5p*, *miR-146b-5p*, *miR-208a*, *miR-221-3p* and *miR-381*) and four downregulated miRNAs (*miR-125b-5p*, *miR-193a-3p*, *miR-193b-3p* and *miR-378a-3p*) [76] (Figure 2C). Interestingly, *microRNA-378* is involved in the regulation of different aspects of muscle biology [82]. An inverse relationship between *miR-378-3p* and its predicted target *MBNL2* was also found [76], and a correlation with adaptive response counteracting the decreased bioavailability of MBNL was speculated. The deregulated miRNAs found in DM2 samples were also measured in skeletal muscle biopsies from 16 DM1, and a common deregulation pathway of *miR-193b-3p*; *miR-208a* and *miR-381* was found [76]. Unfortunately, the limited number of DM2 vs. DM1 miRNAs profiling studies makes it very difficult to predict a common miRNAs signature in muscle damage shared in both diseases.

### 5.2. Circulating miRNAs Deregulation in DMs

The invasive nature of muscle biopsies analysis and the variability between obtained results from different types of muscle biopsies have led to direct research on c-miRs as noninvasive biomarkers of muscle-related diseases. Indeed, c-miRs are stably maintained into the extracellular environment and can be analyzed and quantified faster and easier than those from muscle tissue biopsy. Moreover, it is currently known that in response to muscle injury, miRNAs can be released into extracellular fluids including serum and plasma, and this could represent not exclusively a passive leak from damaged muscle but also a biological response in specific physiological and/or pathological contexts [83]. For all these reasons, circulating miRNAs has been recently proposed as diagnostic, prognostic and therapy monitoring biomarkers in diverse muscular dystrophies, including DMs [84,85]. A miRNAs profile in plasma samples from 36 DM1 patients and 36 healthy subjects identified nine deregulated miRNAs, among which eight miRNAs were increased (*miR-133a*, *miR-193b*, *miR-191*, *miR-140-3p*, *miR-454*, *miR-574*, *miR-885-5p* and *miR-886-3p*) and one (*miR-27b*) was decreased (Figure 2C) [71]. Subsequently, the same group [72] validated five out of nine (i.e., *miR-133a*, *miR-140-3p*, *miR-454* and *miR-574*) of the previously tested miRNAs also in a larger DM1 cohort (103 DM1, and 111 age-and sex-matched healthy subjects). This confirmatory study indicates that these miRNAs correlated with the most clinically relevant parameters of DM1 patients (e.g., skeletal muscle strength and creatine kinase values). Moreover, the identified miRNAs were also found to be deregulated in the plasma of a smaller group of 30 DM2 patients (Figure 2C) [72]. On these bases, the *miR-1*, *miR-133a*, *miR133b* and *miR-206* myomiRs expression level was also investigated in sera from 23 DM1 patients compared to 23 controls showing a positive correlation with the progress of muscle wasting in DM1 patient tested (Figure 2C) [73]. The authors also validated the association of the four miRNAs (*miR-1*, *miR-133a*, *miR-133b* and *miR-206*) in sera of a larger patient cohort (63 DM1 vs. 63 healthy individuals) [74]. In accordance with the previous studies by Perfetti et al., [66,67], they found an increase in circulating *miR-140-3p*, *miR-454* and *miR-574* expression levels in DM1 patients, whereas *miR-27b* levels were significantly elevated in serum samples of DM1 patients compared to healthy subjects (Figure 2C) [74]. Interesting information came from a study comparing the expression levels of 11 candidate miRNAs (*miR-1*, *miR-133a*, *miR-148a*, *miR-152*, *miR-206*, *miR-29b*, *miR-29c*, *miR-335*, *miR-365*, *miR-33a* and *miR-7*) in both whole blood and skeletal muscle biopsy samples from the same DM1 patients. This analysis reveals an upregulation of *miR-133a*, *miR-29b*, and *miR-33a* in blood not reflected in skeletal muscle tissue, where a downregulation of *miR-1*, *-133a* and *-29c* was reported. Taken together, these results depict a diverse miRNAs expression profile in DM1 skeletal muscle and whole blood (Figure 2C) [60]. Lastly, in a recent study, the circulating levels of myomiRs (*miR-1*, *miR-206*, *miR-133a* and *miR-133b*) were analyzed in relation to the physical rehabilitation efficiency in DM1 patients [75]. The expression level of myomiRs was first investigated in sera from nine DM1 compared to seven healthy subjects, founding a relevant upregulation of *miR-1* and *miR-206* in all DM1 patients, whereas *miR-133a* and *miR-133b* upregulation was found only in 2 and 1 patients, respectively. The analysis of myomiRs after a 6 week rehabilitation program showed a significant downregulation of all myomiRs in DM1 patients compared to the pretraining levels. Interestingly, the reduction in the expression of circulating myomiRs was positively correlated to an improvement in muscle strength and endurance. Based on these observations, authors suggest that the circulating levels of myomiRs are potential biomarkers to assess the physical rehabilitation efficiency in DM1 patients [75]. Although the abovementioned studies showed some discrepancies in results imputable to differences in sample size, procedures and part of blood analyzed (i.e., plasma, serum and whole blood), an overall deregulated miRNAs profile emerged (Figure 2C). On these bases, c-miRs are candidate prognostic and diagnostic biomarkers for DMs also useful for monitoring a faster evaluation of responsiveness to treatments.

## 6. Clinical Significance of Epigenetic Signatures in DMs

Although patients born with a larger expansion usually present a more severe phenotype than those with a smaller one, the CTG repeat length only partially explains the large phenotypic variability of DM1, suggesting that epigenetics could play a role in the genotype–phenotype correlations. Nevertheless, only few studies investigate the relationship between *DMPK* methylation levels and DM1 clinical subphenotypes, which could improve the prognosis accuracy in genetic counselling. Most of the published papers were focused on the analysis of DNAme as an indicator of CDM1 better than the CTG repeat length [27,35,40]. Indeed, looking only at maternal inheritance, there is a significant correlation between CDM1 and methylation compared to maternal transmission of noncongenital DM1, providing strength for a maternal effect [34]. CpGs methylation has been also strongly associated with childhood- and juvenile-onset forms of DM1 vs. adult and late-onset forms [34]. Based on these observations, DNAme upstream of the CTG repeats in blood DNA has been proposed as a critical marker of maternally inherited CDM1, in addition to repeat length, the age and the clinical state of the mother. However, very little is known about the effect of CpGs DNAme and the mechanisms behind the multi-systemic involvement in DM1 patients, except for the muscular impairment rating scale (MIRS) in a preliminary Italian study [27]. This gap was filled by Legare et al., who analyzed the DNAme levels of the *DMPK* gene in a large cohort of DM1 adult patients in association with muscular and respiratory impairments [42]. They found that DNAme downstream of the CTG array contributed significantly and independently to explain the phenotypic variability of several muscular and respiratory parameters, including grip/pinch strengths, forced vital capacity and maximal inspiratory pressure. Interestingly, this association is not linked to the CTG repeat length and suggests that *DMPK* epivariations alone could help in predicting the respiratory and muscular profile of patients [42]. It has also been hypothesized that DNAme might even allow one to better understand the cognitive dysfunction often reported in DM1 [28]. A recent study investigates, for the first time, the association between *DMPK* blood DNAme levels and cognitive impairment in 115 adult-onset DM1 patients carrying different expansion length with or without VRs [28]. In patients harboring “pure” (CTG)_n_ expansions, blood showed DNAme at baseline which contributed to predict cognitive functions over a 9 year follow-up period, independently from the CTG repeat length. In accordance with previous observations, patients with VRs have a different DNAme profile (higher downstream hypermethylation) leading to effects of similar magnitude and in the same direction as those observed for patients without VRs. Unfortunately, the limited number of patients analyzed (n = 12) makes it not possible to reach statistically significant correlations between DNAme levels and cognitive impairment in this small subgroup of DM1 “atypical”. Overall, data emerging from these studies encourage the quantification of DNAme level as potential biomarker of DM1 disease progression and to establish a more reliable prognosis for the cognitive, muscular, and respiratory dysfunctions. Nevertheless, further investigations including a higher number of patients (with and without VRs) and more CpG sites in the *DMPK* gene are needed to clearly elucidate the functional relationship between DNAme and the DM1 phenotype. Such studies are necessary before strong conclusions can be drawn and translated into the clinics and genetic counselling for DM1 patients. Unfortunately, to date, none tested the association of DNAme of *CNBP* gene and the different DM2 clinical subtypes. We believe that studies in this direction will greatly help to understand the pathomechanism at the basis of the phenotypic differences between DM1 and DM2 patients.

## 7. Concluding Remarks

Extraordinary progresses have been made in recent years in understanding the genetic and molecular basis of DMs, resulting in the development of animal models and therapeutic approaches. In this scenario, epigenetics is an emerging field of study with strong potential for clinical applications, which needs further investigations to clarify varying and sometimes conflicting data. The discrepancy could be attributable to differences in sample size, as DM cohorts are often numerically limited and control groups are very small or absent. In addition, the use of different and sometimes less sensitive detection methods, especially in early studies, could make it difficult to distinguish the true contribution of epigenetic mechanisms. Undoubtedly, a huge gap that needs to be filled is the paucity of data about the DM2 locus, as most of the studies described so far are strictly focused on DM1. We believe that the application of next-generation sequencing (NGS)-based techniques will have a great impact on epigenetic studies in DMs, shedding light on many still unsolved questions. These high-throughput technologies have the potential to unravel the main epigenetic alterations, including DNAme and hydroxymethylation at the 5-position of cytosine (5mC), associated with the C/CCTG expansions. Defining an epigenetic signature of DMs might be predictive of disease onset, progression or severity. Understanding the clinical significance of these mechanisms may serve as a diagnostic and prognostic indicator in genetic counselling in order to improve genotype–phenotype correlations and to develop translational studies from bench to clinic.

## Figures and Tables

**Table 1:** DNAme studies in DM1, DM2 and control (CTR) individuals.

Disease Form	Tissue	Sample Size	Genomic Context (*DMPK* Gene)	Method of Analysis	Analysis Outcome	Reference
CDM1; Adult	Dura mater, skeletal muscle, skin biopsies and white blood cells	30 DM1	Upstream region of (CTG)_n_ repeats, corresponding to the genomic *Sac*I-*Hin*dIII fragment carrying exons 11–15	Methylation-sensitive restriction enzymes digestion	Hypermethylation in intron 12 at restriction sites of *Sac*II and *Hha*I in CDM1 patients	[35]
DM1 foetuses; DM1 adults; Transgenic DM1 mice	Different source	13 DM1 vs. 3 CTRs	CTCF binding sites upstream and downstream of the CTG repeats	Bisulphite-sequencing PCR and methylation-sensitive restriction enzymesdigestion	Hypermethylation of upstream sequences in DM1 individuals. In DM1, mice methylation pattern was present up- and down-stream of the CTG array	[40]
Childhood-onset; Juvenile/adult-onset; CDM1 with uninterrupted CTG expansions; DM1 “atypical”	Whole blood	66 DM1 including 9 patients with VRs vs. 30 CTRs	DNA sequences (including CTCF-1 and CTCF-2) in 5′ and in 3′ end regions of the CTG array	MS-HRM	Hypermethylation of upstream sequences in CDM1 and childhood-onset patients with large uninterrupted (CTG)_n_ expansions, significantly associated with maternal transmission. First evidence that DM1 patients with VRs show a distinctive hypermethylation pattern at 3′ end of the CTG array	[27]
Premutated DMPK alleles containing VRs	Whole blood	Three-generation Italian family	DNA sequences (including CTCF-1 and CTCF-2) in 5′ and in 3′ end regions of CTG array	Pyrosequencing	Absence of an *in cis* effect of the (CCG)_n_ interruptions on the methylation of the DM1 locus	[43]
DM1-Affected hESC Line Collection	hESCs	14 DM1	DNA sequence spanning from exon 11 to the CTG repeats	Pyrosequencing	Marked increase in methylation levels of the expanded allele	[39]
Late-onset; Adult; Juvenile; Childhood	Whole blood	92 DM1 vs. 10 CTRs	Upstream (CTCF1) and downstream (CTCF2) regions	Sanger Sequencing and Massive Parallel Sequencing	DNAme levels of both CTCF sites higher in CDM1 than in non-CDM1 patients	[34]
Adult-onset; DM1 “atypical”	Whole blood	90 DM1 including 8 patients with VRs	CpG sites upstream and downstream of the (CTG)_n_ expansion	Pyrosequencing	DNAme levels upstream of the (CTG)_n_ expansion were correlated with CTG repeat length, and the presence of a VRs was associated with higher DNAme levels compared to pure CTG array	[42]
Adult-onset; DM1 “atypical”	Whole blood	115 DM1 including 12 patients with VRs	Downstream region (no CTCF binding sites) of the (CTG)_n_ repeats	Pyrosequencing	Patients with VRs alleles had distinctive DNAme and cognitive profiles	[28]
Noncongenital DM1	Whole blood	68 DM1 vs. 73 CTRs	Upstream and downstream regions (no CTCF binding sites) of the (CTG)_n_ repeats	Pyrosequencing	Hypermethylation of both upstream and downstream regions	[44]
DM2	Whole blood	72 DM2 vs. 50 CTRs	CpG islands in the 5′ promoter region and in the region 3′ of the [CCTG]_n_ repetitions	Pyrosequencing	No significant differences in the methylation profile between DM2 patients and CTRs	[45]
	Skeletal muscle	7 DM2 vs. 7 CTRs				
	Skeletal muscle	7 DM2 vs. 7 CTRs				

**Table 2:** Deregulated miRNAs in different biological samples from DM1 and DM2 patients.

*miRNA*	Sample Type DM1	Sample Size	Analysis Outcome (Up or Down Regulation)	Reference
*miR-206*	Skeletal muscle (vastus lateralis)	7 DM1 vs. 4 CTR	Up	[64]
*[miR-1; miR-335 miR-33]*	Skeletal muscle (biceps branchii)	15 DM1 vs. 14 CTR	[Up]	[65]
*miR-29b/c*			Down	
*miR-1; miR-7; miR-10*	Skeletal muscle (vastus lateralis, biceps branchii, deltoid)	5 DM1 vs. 3 CTR	Down	[66]
*miR-206*	Skeletal muscle (vastus lateralis)	12 DM1 vs. 6 CTR	Up	[67]
*[miR-1; miR-133a/b]*			[Down]	
*miR-1; miR-133a; miR-29c*	Skeletal muscle (biceps branchii, deltoid, Gastrocnemium)	9 DM1 Vs. 9 CTR	Down	[68]
*miR-1*	Heart	5 DM1 vs. 8 CTR	Down	[69]
*miR-1; miR-133; miR-29b; miR-30b; miR-145; let-7d/g; miR-23a/b; miR-26a; miR-328; miR-365; miR-499; miR-125b; miR-148°; miR-194; miR-27a; miR-22; miR-100*	Heart	8 DM1 vs. 4 CTR	Down	[70]
*[miR-113a; miR-193b; miR-191; miR-454; miR-574; miR-885-5p; miR-886-3p]*	Plasma	36 DM1 vs. 36 CTR	[Up]	[71]
*miR-27b*			Down	
*[miR-1; miR-133a/b; miR-206; miR-140-3p; miR-454; miR-574]*	Plasma	103 DM1 vs. 111 CTR	[Up]	[72]
*miR-27b*			Down	
*miR-1; miR-133a/b; miR-206*	Serum	23 DM1 vs. 23 CTR	Up	[73]
*miR-1; miR-133a/b; miR-206; miR-113a; miR-193b; miR-191; miR-574; miR-885-5p; miR-886-3p; miR-27b*	Serum	63 DM1 vs. 63 CTR	Up	[74]
*miR-1; miR-133a/b; miR-206*	Serum	9 DM1 vs. 7 CTR	Up	[75]
*miR-133a; miR-29b; miR-33a*	Whole blood	10 DM1 vs. 10 CTR	Up	[68]
*miR-1*	Heart	2 DM2 vs. 8 CTR	Down	[69]
*[miR-34a-5p; miR-34b-3p; miR-34c-5p; miR-146b-5p; miR-208a; miR-221-3p; miR-381]*	Skeletal muscle (biceps brachi)	13 DM2 vs. 13 CTR	[Up]	[76]
*miR-125b-5p, miR-193a-3p, miR-193b-3p and miR-378a-3p*			Down	
*miR-1; miR-133a/b; miR-206; miR-140-3p; miR-454; miR-574*	Plasma	30 DM2 vs. 111 CTR	Up	[72]

## Data Availability

The data presented in this study were extracted from the articles cited in the text.
